# Binocular Function in Different Gaze Positions

**DOI:** 10.18502/jovr.v17i2.10792

**Published:** 2022-04-29

**Authors:** Amir Asharlous, Asgar Doostdar, Vahid Ghaemi, Mina Farzi, Abbasali Yekta, Abolghasem Mortazavi, Hadi Ostadimoghaddam, Mehdi Khabazkhoob

**Affiliations:** ^1^Rehabilitation Research Center, Department of Optometry, School of Rehabilitation Sciences, Iran University of Medical Sciences, Tehran, Iran; ^2^Noor Research Center for Ophthalmic Epidemiology, Noor Eye Hospital, Tehran, Iran; ^3^Department of Optometry, School of Paramedical Sciences, Mashhad University of Medical Sciences, Mashhad, Iran; ^4^Sina Hospital, Department of Neurosurgery, Tehran University of Medical Sciences, Tehran, Iran; ^5^Refractive Errors Research Center, Mashhad University of Medical Sciences, Mashhad, Iran; ^6^Department of Basic Sciences, School of Nursing and Midwifery, Shahid Beheshti University of Medical Sciences, Tehran, Iran

**Keywords:** Accommodation, Binocular Vision, Convergence, Facility, Gaze; Phoria

## Abstract

**Purpose:**

To evaluate varied aspects of binocular function in multiple gaze positions.

**Methods:**

In 2018, this cross-sectional study was conducted on 21 participants (male = 11) with an age range of 19–25 years. Having emmetropia and 10/10 visual acuity in both eyes were conditions of the inclusion criteria for the cross-sectional study. The following aspects of binocular function including amplitude of accommodation (AA), near point of convergence, near phoria, and monocular accommodative facility were evaluated in five gazes (primary, upward, downward, left, and right) for all subjects.

**Results:**

Near point of convergence values showed significant differences in all gaze positions (*P* < 0.001). The lowest near point of convergence value was seen in the primary gaze (2.69 cm) and the downward gaze (3.47 cm) and the highest near point of convergence value was seen in the left gaze (7.5 cm). There was also a significant difference in the amplitude of accommodation among the upward, downward, and the primary gaze (*P* < 0.001) positions but no difference was observed among the temporal, nasal, and the primary gaze positions. There was a significant difference in near phoria between the upward gaze and the primary gaze (*P* = 0.008) while no significant differences were observed among the other gazes. There was no significant variance in the monocular accommodative facility among the different gaze positions (*P* = 0.175).

**Conclusion:**

The results of this study indicated variations that exist in the convergence and accommodation reflex functions in multiple gaze positions, which proved to be more prominent in the convergence system. Although the accommodative sufficiency evaluation was inconsistent among the multiple gaze positions, the accommodative facility evaluation was consistent in all gazes.

##  INTRODUCTION

Binocular vision incorporates several reflexes including accommodation and convergence. Accommodation is a conditional reflex that provides a clear retinal image at different distances through regulating the crystalline lens curvature.^[1]^ Convergence is another conditional reflex of the visual system that is stimulated by disparity on the retina. It occurs following diplopia resulting from stimulation of non-corresponding points in the fellow eyes and preventing diplopia through the disconjugate movement of the eyes.^[2]^ Therefore, convergence is an anti-diplopia reflex while accommodation is an anti-blur mechanism. These two reflexes cooperate in forming a single and clear image at different distances. Suppression or stimulation of the accommodative reflex cause changes in the vergence reflex and vice versa. Therefore, these reflexes have important codependent interactions where disorders in either one can disturb the function of the other.^[3]^

As a result of the multiple gaze positions, especially in upward and downward gazes, vertical rectus, horizontal rectus, and oblique muscles are involved in abduction and adduction processes, so the amount of convergence may vary at different directions.^[4]^ The presence of deviations with A, V, X, and Y patterns indicates that within these deviations, varied amounts of convergence in upward and downward gazes results in diverse amounts of esotropia and exotropia in multiple directions.^[5,6,7]^ Since the accommodative system has a neural link with the vergence system and there is interaction between them, it is expected that the accommodative function will vary in multiple gaze positions, especially in the vertical direction. A few studies have investigated accommodation and convergence variances in multiple gaze positions in the past.^[8,9,10,11]^ Ripple et al reported marked differences in AA in varied gaze positions,^[8]^ while Atchison et al found no significant variances in this regard.^[9]^

As for convergence, a study by Sheni et al on nine subjects found multiple values for convergence in varied gaze positions.^[10]^ The results of an interesting study by Nguyen et al in 2008 showed changes in the accommodative convergence/accommodation results when tested in different gaze positions.^[12]^

As mentioned previously, there were discrepancies in the results of previous studies which suffered from serious shortcomings and flaws. One of the main flaws of these studies was that they only assessed the accommodative or convergence system exclusively and did not evaluate both of them and their codependent effects. In addition, the sample size was very small in some studies like the one conducted by Nguyen et al. Another shortcoming was that they only addressed the amplitude of accommodation and did not study other functional aspects like facility. Therefore, it is recommended that comprehensive studies be conducted to assess the accommodative system from both aspects of amplitude and facility in multiple gaze positions while also comparing the convergence amplitude in the same gaze directions. In addition, besides these two reflexes, the amount of phoria should also be investigated in multiple gaze positions. Considering the strong interaction among these parameters, such studies help to understand their impact on the mechanism of binocular vision in varied gaze positions. This study was conducted to evaluate the binocular function aspects of accommodative and convergence systems as well as phoria in multiple gaze positions.

##  METHODS

This cross-sectional study was conducted on a group of volunteers aged 19–25 years who met the inclusion criteria in 2019. The inclusion criteria were the following: a corrected visual acuity of 10/10 in both eyes; hyperopia, myopia, and astigmatism of maximum 0.5 diopter; complete ocular health; lack of strabismus; and no history of strabismus surgery. The tenets of the Declaration of Helsinki were observed in this study and its protocol was approved by the Ethics Committee of Shahid Beheshti University of Medical Sciences. The participants were assured of data anonymity and confidentiality and informed consent was obtained from all of them.

### Primary Examinations

Demographic data of the participants were recorded and history of ocular and visual problems was taken. All examinations were conducted by an expert optometrist. Auto refraction was done for both eyes to evaluate the inclusion criteria (TOPCON, KR-8900, Japan) and the visual acuity of the fellow eyes was measured using a Snellen chart. Ocular health was then evaluated using a slit lamp (TOPCON SL-6E, Japan). The patients were admitted into the study if they met the inclusion criteria.

### Binocular Examinations

All subjects who met the inclusion criteria and joined the study underwent complete binocular examinations including the measurement of accommodative amplitude, near point of convergence, monocular accommodative facility, and near-cover test to measure near phoria in the primary gaze as well as the upward, downward, left, and right gazes. Positioning for the test target was determined relative to the primary gaze where the visual target was presented at an angle of 40º in each of the various gaze positions.

**Figure 1:**
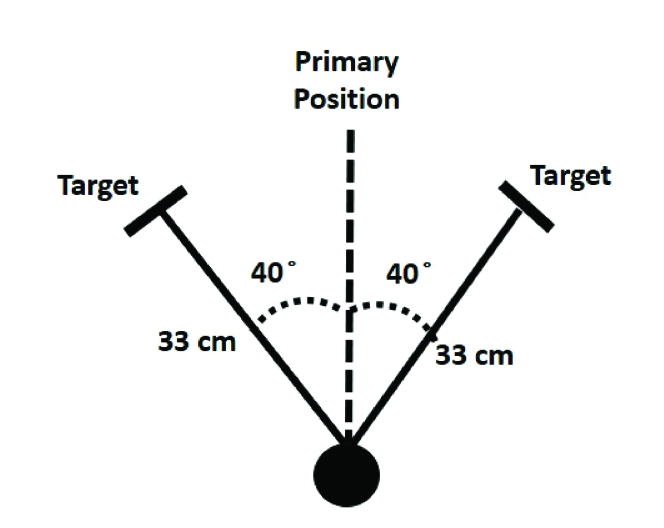
How to adjust angles and distances for measurements at different gaze positions. Cm; centimeter.

A protractor and ruler were used to fix the angle at the different gaze positions. Using a protractor and placing the zero degree on the primary position (facing the patient's pupil), 40º to the relevant gaze was determined and then a 33-cm long ruler was placed from the center of the protractor and at a 40º angle position in the direction of the respective gaze. The target was then placed at the end of the ruler, in the direction of the center of the cornea, and the angle and distance for each test were checked again. All these measurements were done very carefully. In all of the studied gazes, great emphasis was placed on the correct determination of the 40º angle. How to control the angle and distance is illustrated in Figure 1.

The order of the tests was randomized within the various subjects and also within the multiple gaze positions. A 5-min washout time was applied between each test and measurements in the varied gaze positions.

The optotypes on the 20/30 line of a near Snellen chart were used as the fixation target. One eye was occluded and the patient was asked to fixate on the target during the test and try to keep it single and clear. Negative lenses were then added at 0.25 diopter steps. When the target became clear, stronger lenses were introduced. This process continued until the patient reported a sustained blur and could not see the target clearly using the last applied lens. In this condition, the immediate previous lens' power was recorded as the final lens power. The final lens power was then added to 2.5 diopter and the result was recorded as the amplitude of accommodation. This process was done in all of the five gaze positions.

The optotypes on the 20/30 line of a near Snellen chart were also used as the fixation target for the near point of convergence measurement. With both eyes open, the target was moved towards the patient's eyes along the nasal bridge at a speed of 1 cm/s and the patient was asked to keep the target single and clear. The nearest distance between the target and the nasal bridge at which the patient reported a sustained double vision (subjective) or the examiner noticed an ocular deviation or a fusion break (objective) was considered the near point of convergence and its value was recorded in cm. In addition to the primary gaze position, this measurement was repeated in the other four gaze positions at an angle of 40º.

The monocular accommodative facility was measured using ± 2.00 diopter flippers. The 20/30 optotypes were presented to the patient at 40 cm and the patient was asked to fixate on the target during the test and try to keep it single and clear with one eye closed. Positive and negative lenses were alternately presented to the patient and the patient was asked to report the moment the target became clear. A stopwatch was used to measure the time in seconds from the moment the first lens was applied. Each instance of clearing both plus and minus lenses was counted as one cycle, and the number of cycles per 1 min was recorded as the monocular accommodative facility.

An alternate cover test was applied to measure near phoria. Ocular recovery movements were evaluated upon alternate occlusion and the direction of deviation was determined using the recovery direction. A prism bar was then used to measure the amount of deviation using the prism alternate cover test. Corrective prism was added until no recovery movements were noticed on the alternate cover test. Exophoria and esophoria were recorded as minus and plus values, respectively.

### Statistical Analysis

The SPSS software was used for statistical analysis. Mean and standard deviation were used to describe the data.

For statistical comparison, data normality was checked using the Kolmogorov–Smirnov test. To compare the five gazes, the mean values of the gazes were used. To evaluate the difference in variables between males and females, *t*-test was used if the data had a normal distribution and Mann–Whitney was applied if the data had a non-normal distribution.

For analytical analysis, because the data were dependent variables, repeated measured analysis of variance was applied for comparison between the different gaze positions. *P*-values < 0.05 were considered significant.

**Table 1:** The mean and standard deviation (SD) of indices in different gazes according to sex


	**Gaze**	**Male**	**Female**	***P*** -value*	***P*** -value**
	**Mean ± SD**	**Mean ± SD**	* *	* *
Phoria (PD)	Primary	–4.00 ± 3.69	–2.4.0 ± 2.8	0.118	0.28
		Up	–4.91 ± 3.81	–4.00 ± 3.16	0.2	0.561
		Down	–3.18 ± 5.47	–1.00 ± 2.00	< 0.001	0.426 ${}^{***}$
		Left	–4.36 ± 3.44	–2.8 ± 3.26	0.015	0.197 ${}^{***}$
		Right	–3.73 ± 2.83	–2.60 ± 3.13	0.006	0.426 ${}^{***}$
AA (D)	Primary	9.45 ± 1.46	9.40 ± 1.54	0.056	0.934
		Up	9.09 ± 1.58	9.30 ± 1.44	0.016	0.918 ${}^{***}$
		Down	9.91 ± 1.56	9.75 ± 1.57	0.152	0.819
		Left	9.73 ± 1.46	9.35 ± 1.36	0.2	0.547
		Right	9.32 ± 1.35	9.20 ± 1.49	0.2	0.851
NPC (cm)	Primary	22.73 ± 17.94	31.50 ± 16.17	0.044	0.152 ${}^{***}$
		Up	73.18 ± 31.01	60.50 ± 31.57	0.2	0.365
		Down	26.82 ± 20.03	43.50 ± 18.86	0.123	0.065
		Left	67.73 ± 33.86	83.00 ± 27.71	0.2	0.275
		Right	74.09 ± 50.59	66.00 ± 29.42	0.2	0.664
MAF (CPM)	Primary	13.18 ± 8.32	7.10 ± 5.63	0.2	0.067
		Up	10.82 ± 7.19	8.00 ± 7.13	0.2	0.379
		Down	12.00 ± 6.93	7.80 ± 7.05	0.189	0.185
		Left	12.09 ± 7.20	7.90 ± 6.64	0.161	0.183
		Right	12.64 ± 7.42	8.80 ± 7.58	0.159	0.256
	
	

**Table 2:** The mean and standard deviation (SD) of indices in different gazes


	**Left**	**Right**	**Down**	**Up**	**Primary**	***P*** -value ${}^{*}$ * *	***P*** -value ${}^{**}$ * *
	**Mean ± SD**	**Mean ± SD**	**Mean ± SD**	**Mean ± SD**	**Mean ± SD**	
AA (D)	9.54 ± 1.38	9.26 ± 1.38	9.83 ± 1.52	9.19 ± 1.47	9.42 ± 1.46	0.2	0.001
NPC (cm)	7.50 ± 3.13	7.02 ± 4.10	3.47 ± 2.08	6.71 ± 3.11	2.69 ± 1.79	0.2	0.001
MAF (CPM)	10.10 ± 7.09	10.81 ± 7.56	10.00 ± 7.14	9.48 ± 7.13	10.29 ± 7.64	0.099	0.175
Phoria (PD)	–3.62 ± 3.36	–3.19 ± 2.96	–2.14 ± 4.29	–4.48 ± 3.45	–3.24 ± 3.21	0.2	0.026
	
	

##  RESULTS

In this study, the data of 21 subjects, including 11 men (mean age = 21.90 ± 1.78 years) were analyzed. The mean and standard deviation of the parameters in five gaze positions are presented in Table 1 and Table 2 according to sex and all cases, respectively. None of the studied parameters were significantly different between male and female [Table 1].

There was a significant variance in the amplitude of accommodation among the different gaze positions (*P* < 0.001). The results indicated a significant difference in the amplitude of accommodation among the upward and downward gaze positions and the primary gaze position (*P* = 0.002), while there was no significant difference among the left (*P* = 0.261) and right (*P* = 0.149) gaze positions and the primary gaze position.

There was a significant variance in near point of convergence among the different gaze positions (*P* < 0.001). Pairwise comparison between each type of gaze and the primary gaze position indicated significant variances in all gazes (*P* < 0.018). The smallest and largest near point of convergence value was seen in the primary and left gaze position, respectively. Repeated measured Analysis of Variance (ANOVA) showed a significant variance in the amount of near phoria among the five gaze positions (*P* = 0.026), and post-hoc revealed that the difference between the primary gaze and the up-gaze position (*P* = 0.008) showed the highest amount of exophoria.

There was no significant difference in the monocular accommodative facility among the different gaze positions (*P* = 0.175).

##  DISCUSSION

This study compared multiple functions of binocular vision among different gaze positions. As mentioned earlier, there was a significant variance in the near point of convergence among the varied gaze positions. These findings were exactly similar to the results of a study by Sheni et al^[10]^ that reported the minimal vergence amplitude in 40º left and right gaze positions and the maximal vergence range between 10º and 20º below the primary horizontal plane. According to their study, the vergence range increased from the straight-ahead gaze position to 20º below the primary horizontal plane and then decreased from 20º to 40º in the same direction such that the vergence value was smaller in 40º below the horizontal plane as compared to the primary gaze.^[10]^

In addition, near point of convergence findings showed convergence conditions in multiple degrees of gaze as compared to the primary position of gaze in the horizontal plane, while it was not true for vertical gaze positions. Therefore, it can be stated that in the various horizontal gaze positions, the function of the horizontal rectus muscles, including medial rectus and lateral rectus, decrease symmetrically in the left and right gaze positions. It seems that the muscle force of lateral rectus and medial rectus is similar to the degrees of the right and left gaze position. However, this functional symmetry is not seen in the vertical plane. Convergence decreased in the upward and downward gaze positions as compared to the primary position of the gaze, which was more noticeable in the up-gaze position. The reason for this finding may be that horizontal rectus muscles do not apply a similar muscle force in varied degrees of the vertical gaze, which was reported in previous studies as well. Previous studies also found that the muscle force of LR and MR decreased in the extreme up and down gaze positions as compared to the primary gaze. ${}^{\left[13,14\right]}$ Another reason for decreased convergence power in up- and down-gaze positions may be the interaction of superior and inferior oblique muscles in these positions. Since these muscles are both abductor muscles, this abduction decreases convergence in the up-gaze position due to the function of the inferior oblique muscle and in the down-gaze position due to the function of the superior oblique muscle.^[15]^

The question is why is convergence stronger in the downward gaze as compared to the upward gaze position? The first reason may be the conditional nature of the vergence reflex.^[16]^ Convergence is a conditional reflex, that is, it is capable of learning and improving over time. ${}^{\left[16,17\right]}$ It can be concluded that more ocular work in the down-gaze position during life due to normal activities like reading and other near-work activities, which are usually done in this gaze position, strengthen the vergence reflex in the down-gaze position as compared to the up-gaze position. This is also consistent with the results of a study by Sheni that reported the maximal vergence range between 10º and 20º below horizontal plane, which is the gaze position used for reading and other near-work activities, ${}^{\left[18,19\right]}$ as compared to the primary position of the gaze.^[10]^ The second reason may be related to the neural link of the vergence and accommodative systems, that is, the ratio of accommodative convergence to accommodation.^[20]^ Since the results of this study and some previous studies showed a stronger accommodative reflex in the down-gaze versus the up-gaze position, ${}^{\left[8,9\right]}$ it can be expected that the same may be true for convergence as well. In fact, because there is more accommodation in the down-gaze position, accommodative convergence is also stronger, resulting in higher amplitude of convergence.

The accommodative findings of the present study revealed the largest accommodative power in the down-gaze and the smallest in the up-gaze position. Some previous studies found similar results. Ripple et al found that the largest amplitude of accommodation was for the down-gaze position followed by the nasal gaze.^[8]^ However, the dioptric amount of difference was much larger in their study as compared to the present study such that the amplitude difference was 3 diopters between the extremes of the vertical plane and 1.5 diopter between the extremes of the horizontal plane. Atchison et al found a higher amplitude of accommodation at the down-gaze position as compared to other directions and reported that this difference, although clinically unimportant, was statistically significant.^[9]^ Their findings are consistent with our results. In general, as for amplitude of accommodation changes in different gaze positions, it can be stated that accommodation usually has slightly larger amplitude in the primary and down-gaze positions and is weaker in the up-gaze position. A relatively stronger accommodation in the down-gaze position may be due to the conditional nature of the accommodative reflex. Accommodation is also a conditional reflex and repeated usage of conditional reflexes during life strengthens them.^[16]^ It may seem logical that because routine near-work activities are done in the down-gaze position, this reflex is strengthened in this position and because the use of accommodation is very limited in the up gaze, its amplitude is not as large.

Regarding phoria, only the difference in near phoria between the up-gaze and primary gaze positions was significant, and this gaze had the largest amount of exophoria. The highest and lowest amount of exophoria was seen in the up- and down-gaze positions, respectively, while there was no significant difference in the amount of exophoria between left- and right-gaze positions and the primary position of gaze. These findings were consistent with the results of near point of convergence. A larger range of vergence in the down-gaze and a smaller range in the up-gaze position contribute to more exophoria in the up-gaze position. On the other hand, there was no difference in near point of convergence between the left- and right-gaze positions and the primary position of the gaze, which was similar to exophoria. An even more interesting finding was that near point of convergence was slightly larger in the left gaze versus the right gaze (which was not statistically significant), indicating a stronger convergence in the right gaze. This finding was consistent with the results of phoria in the resent study, indicating lower exophoria in the right-gaze position.

Although there were significant differences in amplitude of accommodation and vergence range between different gaze positions, no significant difference was found in accommodative facility. Therefore, it can be concluded that amplitude of accommodation has no marked relationship with facility from a functional point of view and they have separate functions, which emphasizes that different aspects of the accommodative function should be evaluated separately and their findings should be analyzed independently in the clinical setting.

Based on the results of the present study, it can be concluded that amplitude of accommodation and convergence are inconsistent in multiple gaze positions. This difference is more apparent in the vertical plane in the accommodative system and in both the vertical and horizontal planes in the vergence system. Convergence decreases symmetrically from the primary position of gaze toward left- and right-gaze positions (horizontal plane) while its changes are completely asymmetrical in the vertical plane with a larger decrease in the up-gaze position. Although amplitude of accommodation varies in different gaze positions, there is no variance in accommodative facility among the different gaze directions. Phoria changes were similar to and consistent with convergence changes in various gaze positions: the highest and lowest amount of exophoria was seen in the upward and downward gaze positions respectively, while there was no significant difference in the amount of exophoria between the horizontal gaze positions and the primary position of gaze.

##  Financial Support and Sponsorship 

This project was supported by Iran University of Medical Sciences.

##  Conflicts of Interest

No conflicting relationship exists for any author.
